# Bone union of the transferred coracoid graft is the key factor affecting the extent of postoperative graft changes and the clinical results following the modified Bankart and Bristow procedure: a computed tomography scan study

**DOI:** 10.1186/s13018-019-1129-6

**Published:** 2019-03-21

**Authors:** Takeshi Makihara, Masayuki Abe, Masashi Yamazaki, Kenji Okamura

**Affiliations:** 1 Department of Orthopaedic Surgery, Faculty of Medicine, University of Tsukuba, 1-1-1 Tennnodai, Tsukuba, Ibaraki 305-8575 Japan; 2 Hitsujigaoka Hospital, 3-1-10 Aoba, Atsubetsu, Sapporo, Hokkaido 004-0021 Japan

**Keywords:** Osteogenesis, Bone resorption, Bristow procedure, Computed tomography, Coracoid graft

## Abstract

**Background:**

The extent of postoperative changes in the coracoid process grafted during the modified Bankart and Bristow procedure remains unclear. The purpose of the present study was to quantify the postoperative changes in bone surface area as assessed on computed tomography, as well as to clarify the impact of such changes on the clinical results.

**Methods:**

Twenty-three shoulders of 21 subjects who underwent the modified Bankart and Bristow procedure were retrospectively analyzed. Computed tomography images were obtained immediately after surgery and at the final follow-up. The changes in bone surface area of the grafted coracoid process were measured on computed tomography slices in the proximity of the screw bore. Clinical outcomes were evaluated in terms of the Rowe, Walch-Duplay, and simple shoulder test scores.

**Results:**

Bone area increased in 15 shoulders (65.2%) and decreased in eight shoulders (34.8%). Bone area increased by 51.3% in shoulders with bone union in the superior part of the coracoid process graft, with no significant differences between the superior and inferior sides of the graft regarding the rate of change in bone surface area (41.4% vs. 68.9% increase). However, in shoulders with bone union in the inferior part of the coracoid process graft, the rate of change in bone area differed significantly between the superior and inferior sides of the graft, exhibiting a 42.3% decrease on the superior side and 39.8% increase on the inferior side. In shoulders with no bone union, bone area decreased by 29.5% (17.4% vs. 39.3% decrease on the superior and inferior side, respectively), whereas the Rowe and Walch-Duplay scores were significantly lower than those noted in shoulders with bone union.

**Conclusions:**

Postoperative bone formation and bone resorption in the coracoid process grafted during the modified Bankart and Bristow procedure depend on whether and where bone union occurs. Graft non-union is associated with inferior clinical results.

## Background

While arthroscopic Bankart repair is the standard procedure used to manage anterior glenohumeral instability, this procedure is associated with high re-dislocation rates in contact sport athletes and in patients with glenoid bone loss [1–7]. In such cases, favorable outcomes can be achieved by the Latarjet and Bristow procedures (both open and arthroscopic), which involve cutting the coracoid process and grafting it on the scapular neck [8–12]. Indeed, recent results of long-term patient evaluations have confirmed that, compared to procedures involving coracoid process transfer, Bankart repair was associated with lower patient satisfaction and higher re-dislocation rates [13, 14].

Procedures using coracoid process grafting can achieve osseous stabilization both via a static effect, which is provided by the coracoid process graft itself, and via a dynamic effect, which is provided by the conjoint tendon and the subscapularis muscle [15]. In the Latarjet procedure, a 2.5–3-cm segment is removed from the coracoid process and then laid lengthwise (i.e., on its side) along the anterior side of the scapular neck, followed by fixation at the top and bottom with two screws. By contrast, in the Bristow procedure, a shorter segment (1 cm in length) is removed from the tip of the coracoid process, placed perpendicularly to the anterior side of the scapular neck, and fixed in place using a single screw. Although these procedures are similar in that they employ the tip of the coracoid process as a graft, biomechanical analysis indicates differences in terms of dynamic movement [16].

In a computed tomography (CT) study, Di Giacomo et al. have reported an extremely high incidence of bone resorption following bone grafting in the Latarjet procedure, as well as variability in the degree of bone resorption in different sections of the coracoid process [17–19]. However, such detailed data regarding the Bristow procedure have not been published to date. As the dynamic movement enabled by the Latarjet procedure differs considerably from that enabled by the Bristow procedure, it is expected that the progression of bone resorption differs between the two procedures. Moreover, the grafting site is comparatively small in the Bristow procedure, and it has a different spatial relationship to the conjoint tendon that supplies blood flow. Based on follow-up evaluations conducted for an average of 17.5 months, Di Giacomo et al. concluded that bone resorption may not affect clinical results [17], but the long-term dynamics of bone resorption remain unclear. Considering the risk of complications associated with resorption-induced screw exposure, the study of bone resorption after the Bristow procedure remains a crucial area of investigation. Similar to the Latarjet procedure, the Bristow procedure is performed worldwide and is the intervention of choice when the length of the coracoid process that can be harvested is too short for the Latarjet procedure to be performed. However, there have been no detailed reports regarding bone resorption after the Bristow procedure.

The aim of the present study was to clarify the dynamics of bone resorption following the modified Bankart and Bristow procedure (BB procedure) by analyzing CT images taken immediately after the procedure and at the last follow-up examination. Additionally, this study aimed to clarify the relationship between the postoperative changes in the coracoid graft and the clinical results of the BB procedure.

## Methods

The study enrolled 23 shoulders of 21 patients followed up for 10 months or longer after the BB procedure. This study was approved by the Institutional Review Board (HH-IRB#16-1), and written consent forms were obtained from all patients. Of the 21 patients included in this study, 16 were men and five were women. The average age in this case series was 21.1 years (age range, 12–38 years).

### Surgical technique

All surgeries were performed by the same surgeon (KO). Surgery was performed via the deltopectoral approach. An incision was made above the superior part of the subscapularis tendon, at 1 cm from the bicipital groove, leaving the joint capsule intact and splitting along the muscle fibers. Approaching through the rotator interval, the joint capsule was separated from the glenoid and mobilized. Then, decortication was performed at the front of the scapular neck. Bankart repair was performed using four or five suture anchors. A 1–1.5-cm segment of the coracoid process was used for grafting. The harvested tip of the coracoid process was reshaped to increase the surface area for the graft. The graft was placed in the 4 or 8 o’clock position (for the right or left shoulders, respectively) and fixed in place using a 3.5-mm cortical screw. The screw was sized so as to penetrate the cortical bone below the graft and inserted as parallel as possible to the glenoid, pointing slightly upward.

### Imaging analysis

#### CT scanning and image reconstruction

**Fig. 1:**
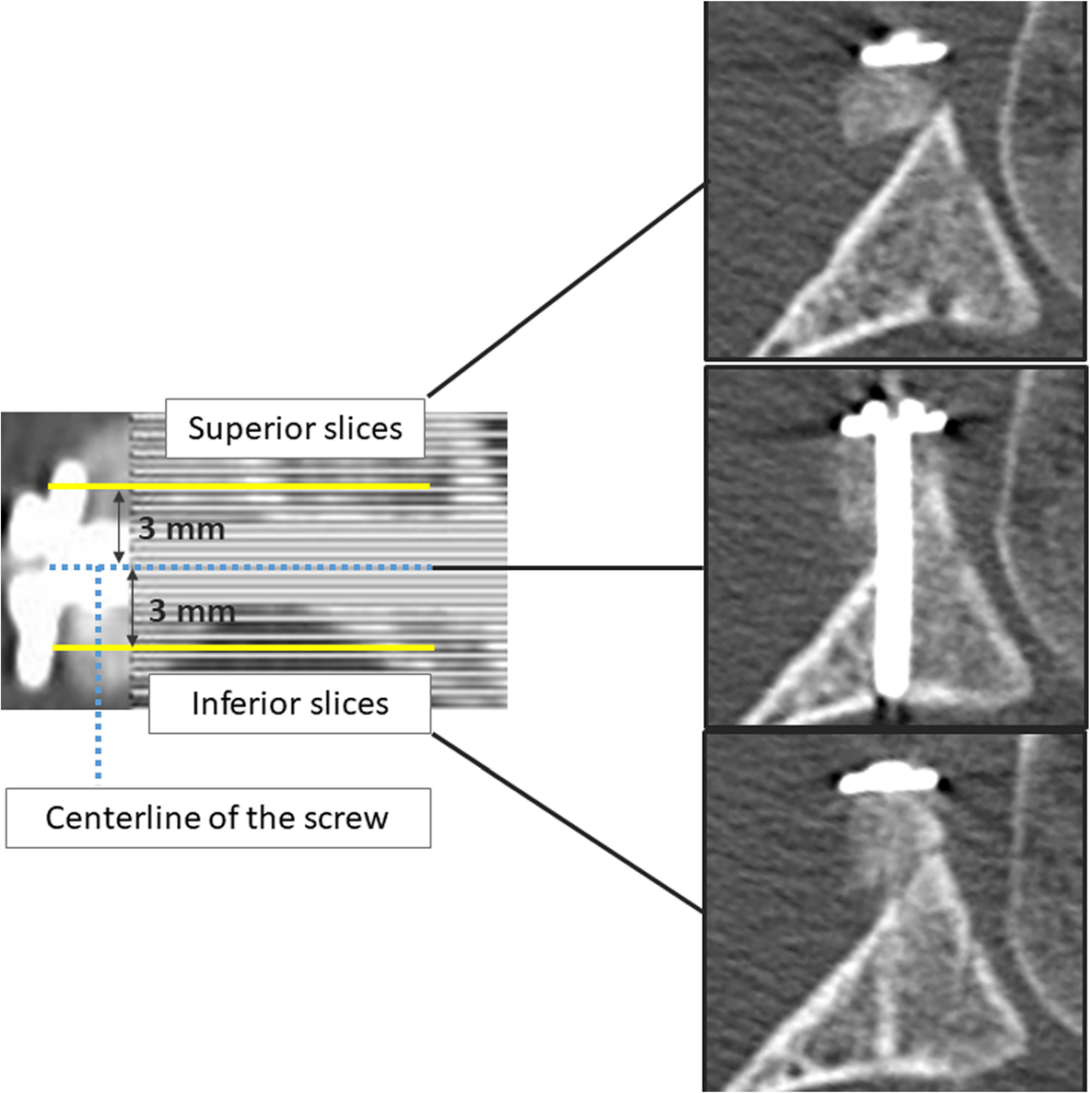
Horizontal cross-sectional view along the length of the screw. At 3 mm above and below the central cross-section of the screw, the computed tomography slices contain views of the superior and inferior parts of the graft, respectively (“superior slices” vs. “inferior slices”)

CT scans were obtained immediately after surgery and at the final follow-up. The acquired volumetric imaging data underwent analytical reconstruction, which was performed using a work station. The scanning angles were chosen such that the horizontal and sagittal cross-sectional views showed the entire length of the screw. In the horizontal cross-sectional view, the scanned volume was divided into slices of 1 mm in thicknesses (Fig. 1). At 3 mm above and below the central cross-section of the screw, which was considered to represent the central cross-section of the coracoid graft in horizontal view, CT slices contained views of the superior and inferior parts of the graft, respectively (“superior slices” vs. “inferior slices”).

#### Radiological evaluation

**Fig. 2:**
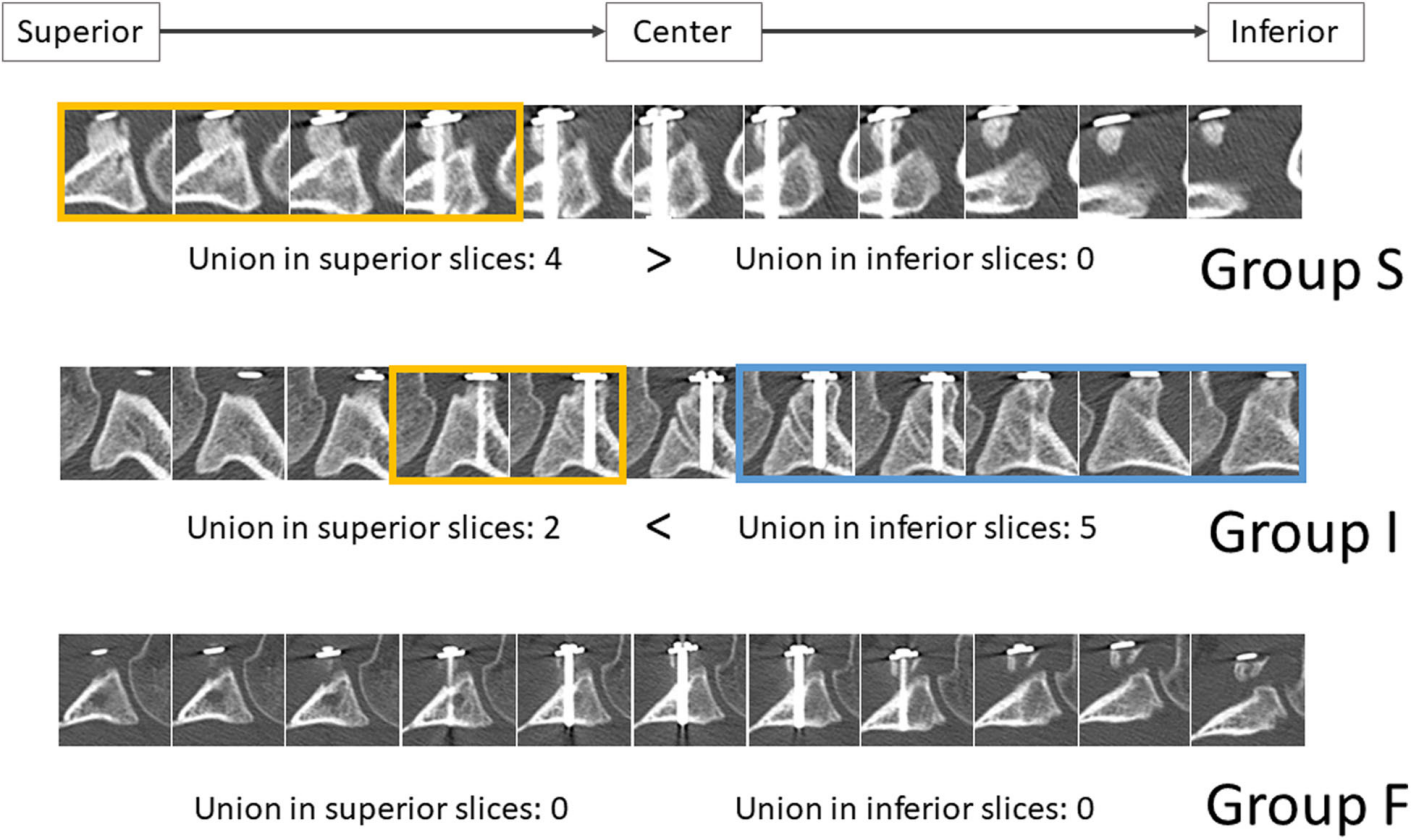
Evaluation of bone union status and location. Shoulders were categorized according to the number of superior and inferior slices in which bone union was visible

The series of horizontal cross-sectional CT slices were examined to identify the slices where bone union between the coracoid process graft and scapular neck was visible. For each shoulder, the number of superior and inferior slices in which bone union was visible was counted. Subsequently, the shoulders were grouped according to the number of CT slices with bone union. Shoulders with bone union predominantly in the superior part of the graft (i.e., visible more in superior slices) were included in group S. Shoulders with bone union predominantly in the inferior part of the graft (i.e., visible more in inferior slices) were included in group I. Shoulders with no bone union (i.e., not visible in either superior or inferior slices) were included in group F (Fig. 2).

**Fig. 3:**
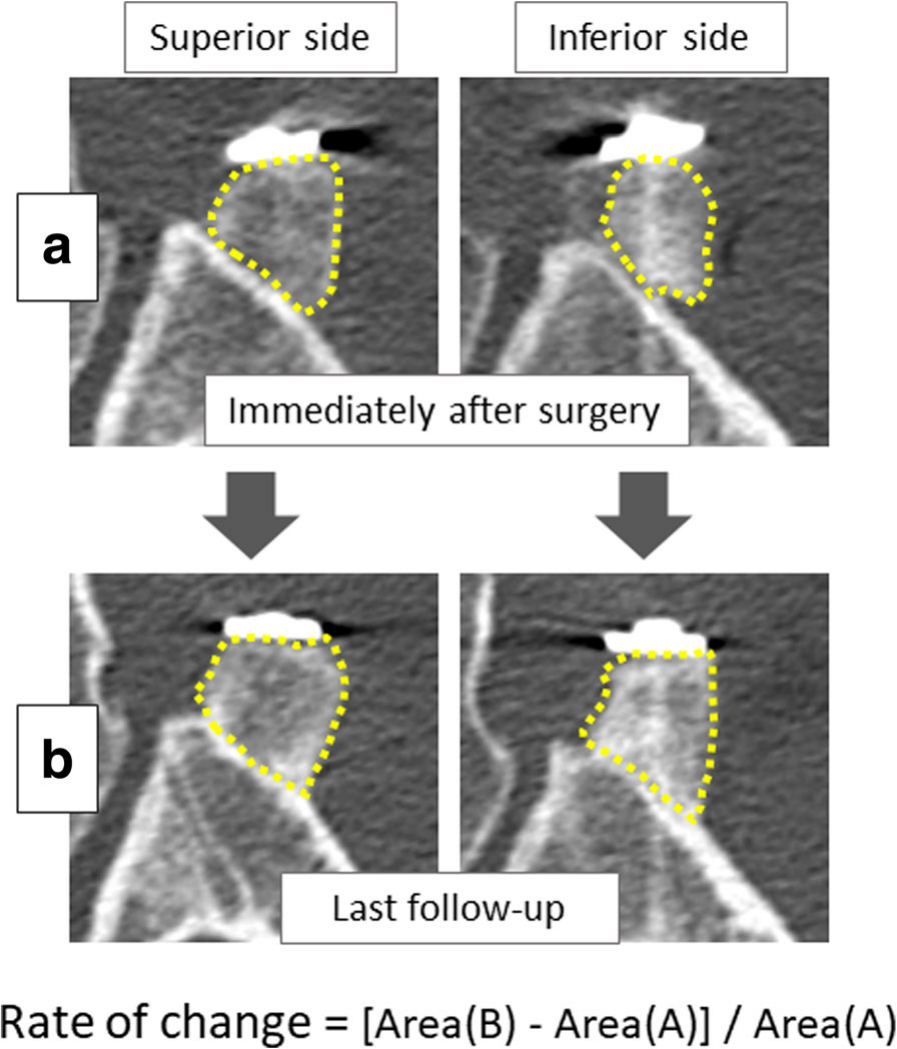
Measurement of changes in the surface area of the graft. Computed tomography scans were taken immediately postoperatively (**a**) and at the last follow-up (**b**). In the most superior and most inferior slices, respectively, the shape of the superior and inferior sides of the coracoid process graft was traced to measure their area. The rate of change in bone area was calculated for each side. The overall change in bone area was also calculated (i.e., averaged for the superior and inferior sides)

In the analytically reconstructed CT slices at 3 mm above and below the screw (most superior and most inferior slices, respectively) (Fig. 1), the outer circumference of the coracoid graft was traced and its surface area was measured using dedicated image analysis software (ImageJ; National Institutes of Health, Bethesda, MD) (Fig. 3). Using the image-processing software GIMP 2 (www.gimp.org), the images taken immediately after surgery were superimposed over those taken at the last follow-up examination, thus clarifying the boundary between the coracoid graft and the scapular neck even after union was achieved. The rate and sign of change in bone area (increases or decrease) were calculated in each group (groups S, I, and F) and for each side of the graft (superior or inferior) by comparing bone area measurements from CT scans taken immediately after surgery against measurements from scans taken at the last follow-up examination (Fig. 3).

### Clinical evaluation

Clinical outcomes were evaluated in terms of the simple shoulder test, Rowe, and Walch-Duplay scores at the last follow-up examination.

### Statistical analysis

Data regarding groups S, I, and F were compared using analysis of variance with a post hoc Tukey-Kramer test, whereas data regarding the superior and inferior parts of the graft were compared using the paired *t* test. A *p* value of < 0.05 was considered to indicate statistical significance.

## Results

**Fig. 4:**
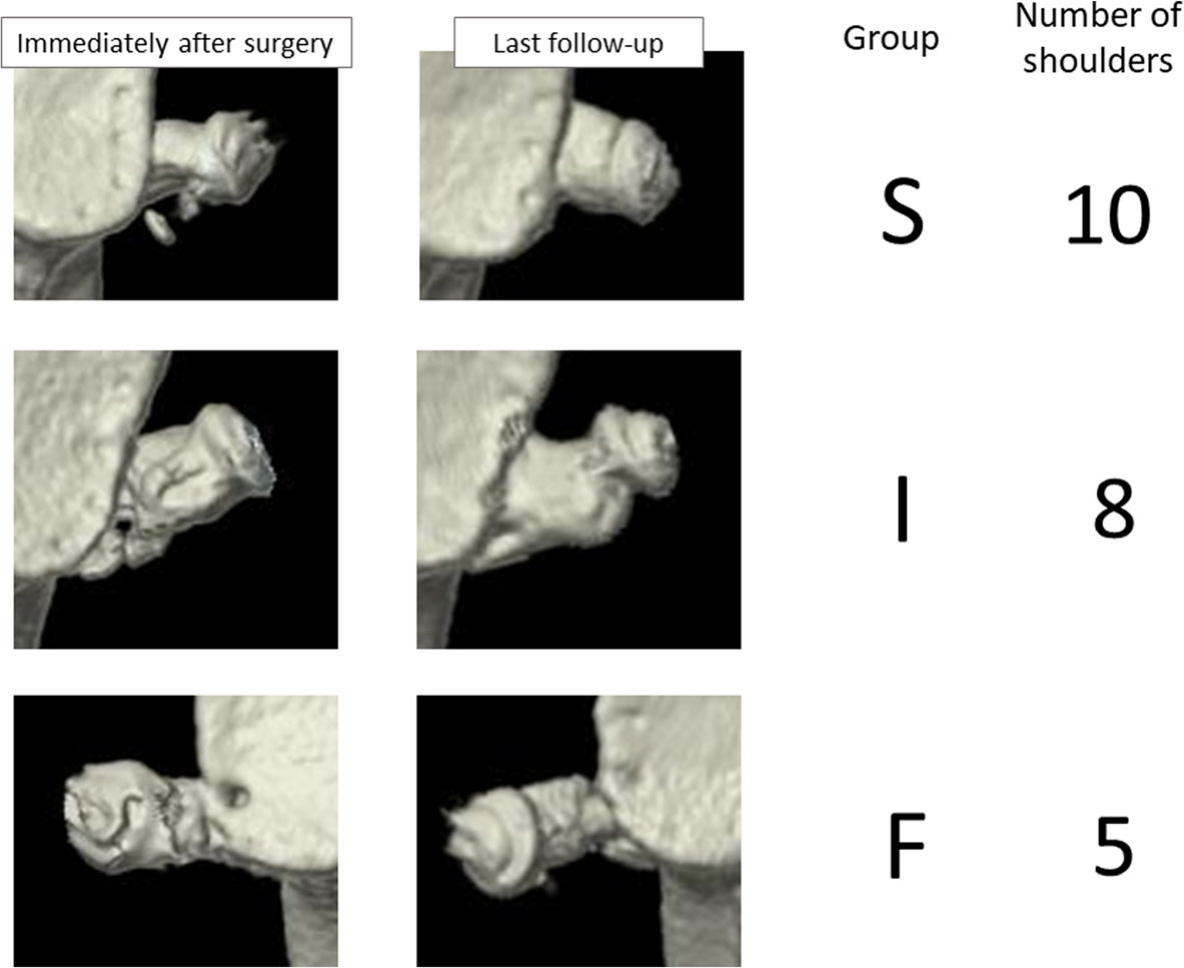
Representative three-dimensional images of the grafted coracoid process. Shoulders were categorized according to the location of bone union, which was achieved mainly in the superior part of the coracoid graft (group S), mainly in the inferior part of the graft (group I), or not at all (group F)

**Fig. 5:**
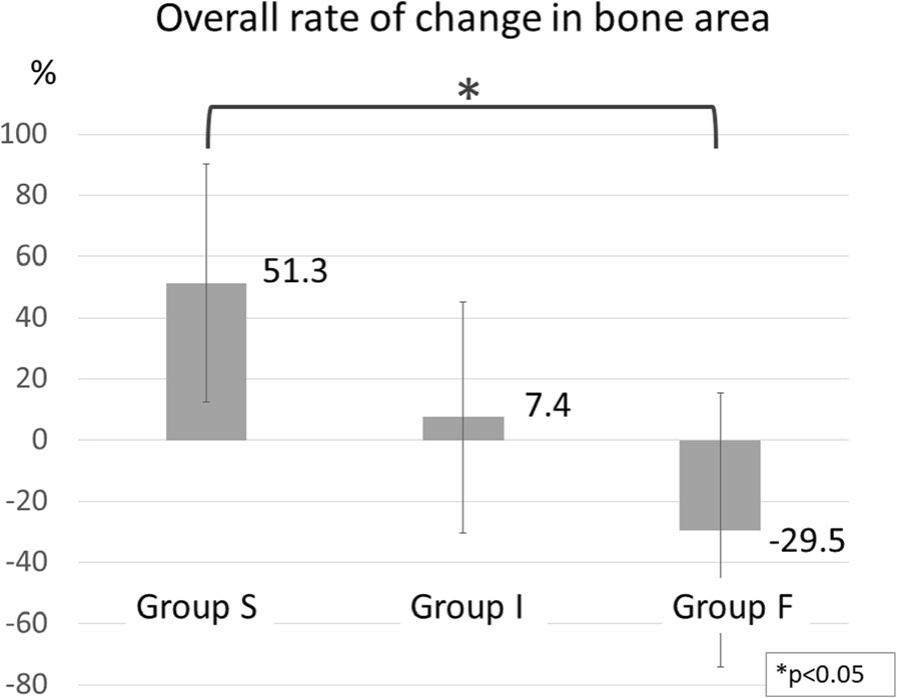
Overall rate of change in bone area (i.e., averaged for the superior and inferior sides of the graft). Shoulders were categorized according to the location of bone union, which was achieved mainly in the superior part of the coracoid graft (group S), mainly in the inferior part of the graft (group I), or not at all (group F). There was a significant difference between groups S and F

**Fig. 6:**
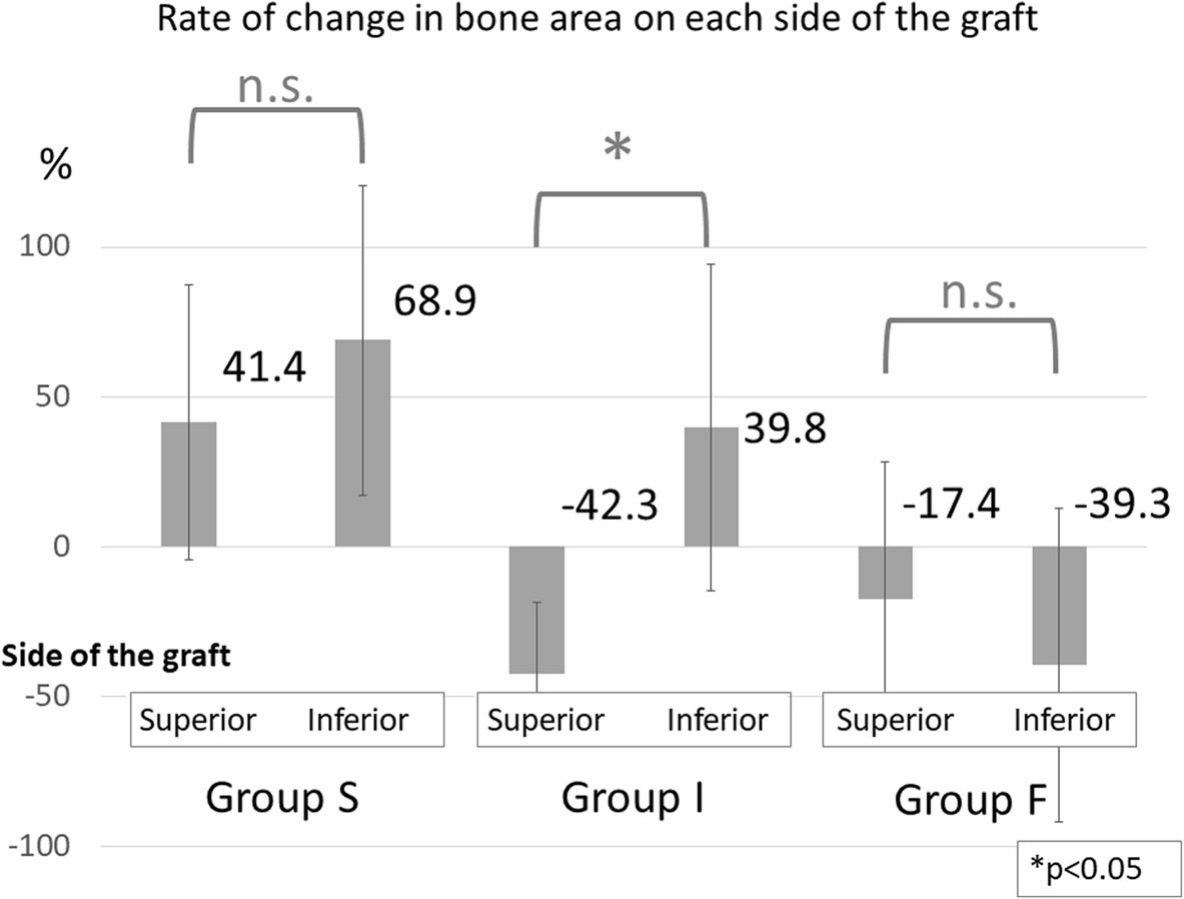
Rates of change in bone area for the superior and inferior sides of the graft. Shoulders were categorized according to the location of bone union, which was achieved mainly in the superior part of the coracoid graft (group S), mainly in the inferior part of the graft (group I), or not at all (group F). In group I, the rate of change differed significantly between the superior and inferior sides. Abbreviations: n.s., not significant

The average follow-up period was 19.2 months (range, 10–32 months). Bone union was noted predominantly in the superior part of the graft in ten shoulders (group S), predominantly in the inferior part of the graft in eight shoulders (group I), and not at all in five shoulders (group F) (Fig. 4). No screw loosening occurred. All screws remained in place until the final follow-up examination. Overall (i.e., for both the superior and inferior sides of the graft), there was an average bone area increase of 18.4%, with 15 shoulders (62.5%) showing a positive change and eight shoulders (34.8%) showing a negative change. The average rate of change in bone area was 53% (11.0 to 129%) for group S, 7.4% (− 21.3 to 85.4%) for group I, and − 29.5% (− 77.2 to 40.9%) for group F, with the difference between groups S and F being significant (Fig. 5). In group S, the average rate of change in bone area was 41.4% (− 23.6 to 147.3%) for the superior side of the graft and 68.9% (13.7 to 171.9%) for the inferior side. In group F, the average rate of change in bone area was − 17.4% (− 85.8 to 36.7%) for the superior side and − 39.3% (− 90.0 to 42.7%) for the inferior side. No significant difference regarding the rate of change in bone area was noted between the superior and inferior sides of the graft in groups S and F. However, in group I, the superior and inferior sides of the graft differed significantly regarding the rate of change in bone area, with − 42.3% (− 70.7 to − 10.6%) for the superior side and 39.8% (− 10.6 to 154.0%) for the inferior side (Fig. 6). The Rowe score was 96 for group S, 95 for group I, and 88 for group F. The Walch-Duplay score was 93 for group S, 91.3 for group I, and 76 for group F. Both Rowe and Walch-Duplay scores were significantly lower in group F than in groups S and F. On the other hand, the simple shoulder test scores did not differ significantly across the groups (98.1 for group S, 98.9 for group I, and 90.9 for group F) (Table 1).

**Table 1:** Clinical outcomes of the modified Bankart and Bristow procedure

Evaluation method	Group S	Group I	Group F
Rowe test score	96 ± 3.9	95 ± 4.6	88 ± 5.7*
Walch-Duplay test score	93 ± 10.6	91.3 ± 9.9	76 ± 11.4*
Simple shoulder test score	98.1 ± 4.1	98.9 ± 3.2	90.9 ± 13.1

A total of 23 shoulders were stratified according to the location of bone union in the grafted coracoid process: superior part (group S), inferior part (group I), or no bone union (group F)

*p* < 0.05 *: significantly lower vs group S, group I (*p*<0.05)

## Discussion

The results of the present study, which employed computed tomography to quantify the postoperative changes in the grafted coracoid process, revealed obvious changes in the graft surface area, which depended on whether and where bone union occurred. In group S, the bone surface area increased for both sides of the coracoid process graft, whereas in group I, the overall rate of change was minimal because the two sides of the graft exhibited rates of change with opposite signs (i.e., decrease in bone area on the superior side and increase in bone area on the inferior side). The finding regarding the effect of bone union location (superior vs. inferior part of the coracoid process graft) on the rate of change in bone area is consistent with the observations of Di Giacomo et al. [17], who found that, following the Latarjet procedure, bone resorption occurred commonly on the superior side of the graft, but rarely on the inferior side. Physical dynamics, graft compatibility, and blood flow were proposed as factors responsible for this variability in bone changes after the Latarjet procedure. The present research shows that, following the Bristow procedure, the sign of the change in surface area depends on the location of bone union. Specifically, while the results for group I resemble those reported by Di Giacomo et al., this is not the case for group S, wherein both the superior and inferior sides of the graft showed an increase in bone area. The relevance of each potential causative factor previously suggested to play a role in bone changes after Latarjet procedure is discussed in further detail below.

**Fig. 7:**
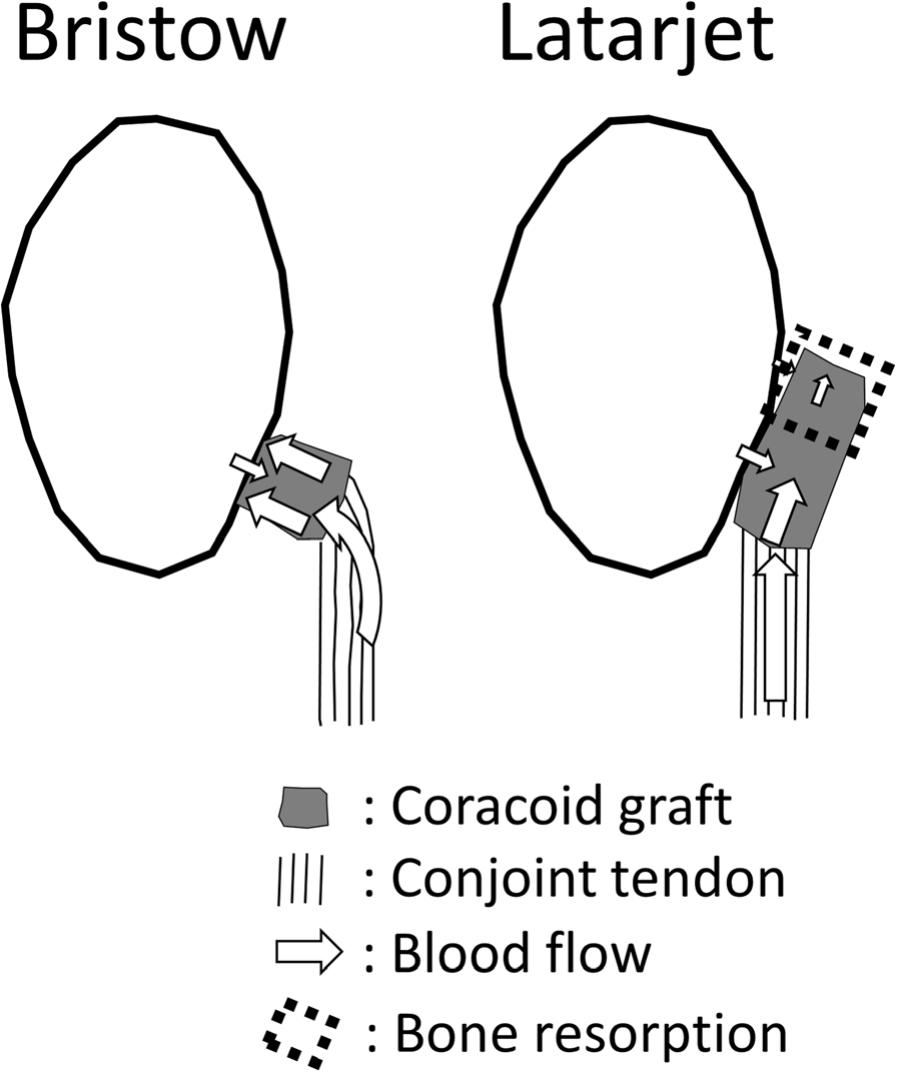
Comparison of the Bristow and Latarjet procedures regarding graft compatibility and blood flow. The superior and inferior parts of the coracoid graft may experience different bone metabolism due to different blood supply (which would depend on the distance from the conjoint tendon) and different stress distribution (which would depend on the graft compatibility with the anterior surface of the glenoid neck). These differences are likely less pronounced in shoulders treated via the Bristow procedure than in those treated via the Latarjet procedure, where the inferior part of the graft is in close contact with the scapular neck and lies close to the conjoint tendon, whereas the superior part lies farther from the conjoint tendon and may be less compatible with the scapular neck

**Fig. 8:**
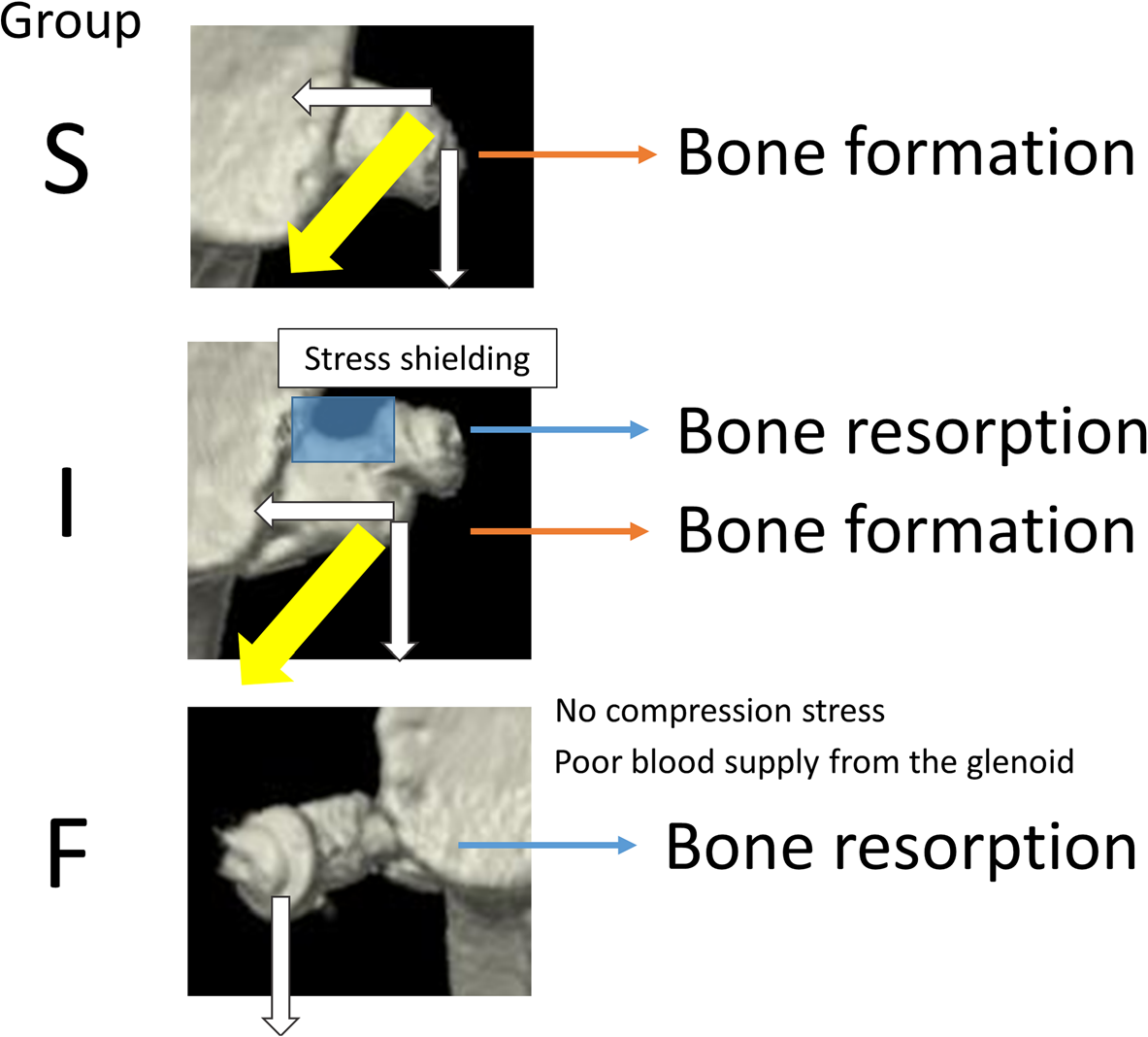
Potential mechanism underlying bone metabolism within the grafted coracoid process. We hypothesize that the dynamic environment of the graft depends on the location of bone union (inferior or superior part of the graft), which enables mechanical stress to be borne by the respective part, promoting bone formation

In terms of graft compatibility, one difference between the Latarjet and Bristow procedures is the area of osteotomy, which is smaller for the Bristow procedure; therefore, after the Bristow procedure, the environments of the superior and inferior parts of the graft are less affected by the curvature of the scapular neck (Fig. 7). The postoperative evolution of the graft can also be assessed in terms of blood flow, which is provided by the conjoint tendon. Specifically, in the Latarjet procedure, the conjoint tendon contacts the inferior side of the coracoid graft, which is believed to restrict blood flow to the superior part of the graft, whereas in the Bristow procedure, the conjoint tendon lies close to both the superior and inferior sides of the graft, suggesting that there is less of a difference in blood flow between the two sides of the graft (Fig. 7). Therefore, it is believed that the differences observed between the superior and inferior sides of the graft could be better explained in terms of the physical dynamics of bone union. Specifically, when bone union occurs in the superior part of the graft, the compression stress of the screw and the pulling stress of the conjoint tendon are both borne by the coracoid process graft as a whole (Fig. 8, top). However, when bone union occurs in the inferior part of the graft, only the inferior part bears the compression and pulling stress, while the superior part is shielded from mechanical stress (Fig. 8, middle). It is thus reasonable to assert that this is the mechanism underlying the change in bone surface area of the graft after the Bristow procedure, namely an increase on both sides for group S, but an increase on the inferior side and a decrease on the superior side for group I. The same mechanism explains the findings for group F, wherein there is no compression stress because of failure to achieve bone union; thus, there is no shared blood flow to the graft, resulting in bone resorption (Fig. 8, bottom).

**Fig. 9:**
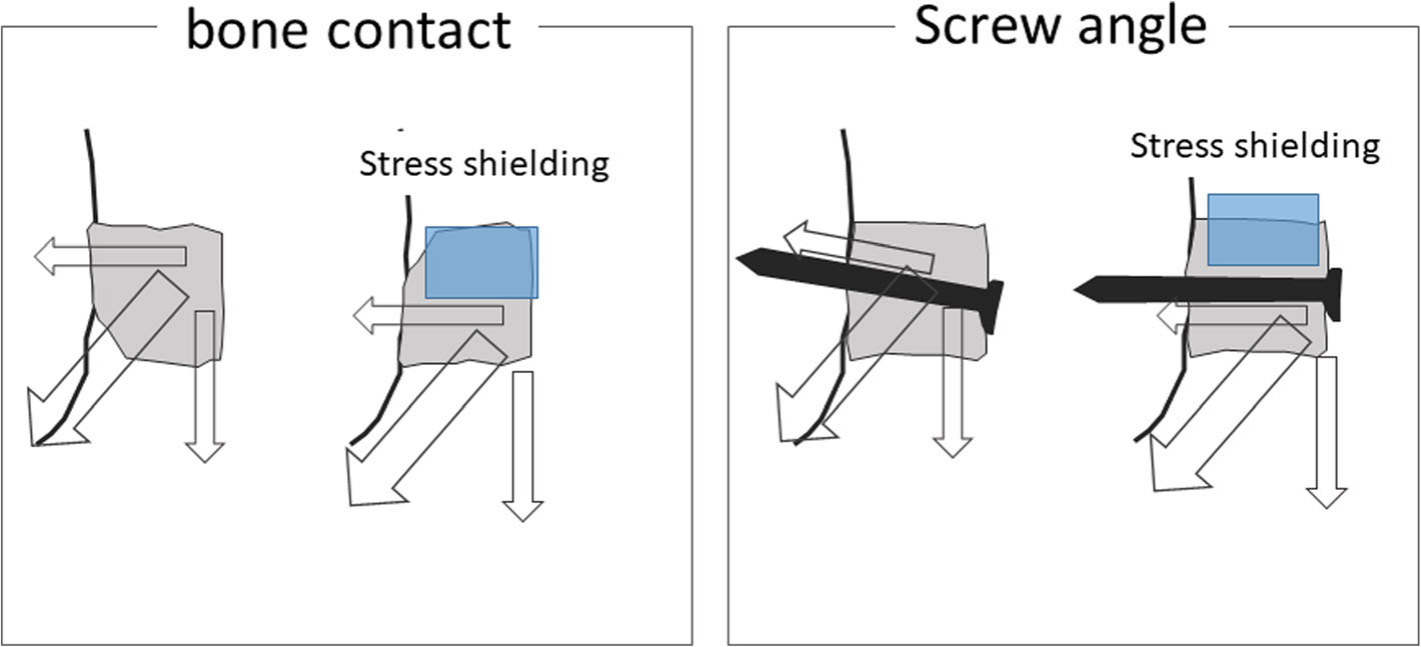
Factors affecting mechanical stress distribution in the grafted coracoid process. Mechanical stress distribution depends on the screw penetration angle and the compatibility between the surfaces of the coracoid process graft and scapular neck. Therefore, increasing the compatibility of the bone surfaces and changing the angle of screw penetration are helpful strategies for modulating the stress borne by the superior side of the bone piece

The results of the present research indicate that, to suppress bone resorption, it is necessary to stabilize the superior part of the graft, thus encouraging bone union in this part. Such bone union can be promoted by shaping the piece of bone to be grafted so that its superior part fits more tightly against the scapular neck. Moreover, screws can be placed so that they penetrate the bone closer to the superior side or inserted at an angle pointing upwards (i.e., toward the superior side of the graft) so that the compression and pulling stresses can be borne by the coracoid process graft as a whole (Fig. 9). Therefore, the findings of the present study can be used when planning graft modeling and screw placement in the Bristow procedure.

Previous studies reported extremely high rates of bone resorption following Latarjet procedures—100% according to Di Giacomo et al. [17] and 90.5% according to Zhu et al. [20]. The present study revealed that, following the Bristow procedure, only eight of 23 shoulders (34.8%) showed a decrease in bone surface area of the graft, whereas the remaining 15 shoulders showed an increase. Examination of the factors potentially responsible for bone resorption (physical dynamics, graft compatibility, and blood flow) suggests that the Latarjet procedure disfavors the superior part of the graft, and the present findings do not contradict this. To the best of our knowledge, no reports have examined bone resorption following the Bristow procedure; therefore, the present study brings new and important information regarding the difference between the Latarjet and Bristow procedures.

Di Giacomo et al. concluded that the Latarjet procedure was associated with bone resorption of the graft, but there appeared to be no effect on clinical outcomes over an average follow-up of 28 months, nor were there any complications resulting from graft resorption [17]. In the present study, bone resorption was observed in groups I and F, leading to screw exposure; however, there was no significant difference in the clinical outcomes between groups S (no bone resorption) and I (some bone resorption), whereas the Rowe and Walch-Duplay scores for group F (substantial bone resorption) were significantly lower than those in group S. If bone union is achieved, the coracoid process graft is stabilized, providing sufficient stability for the forward movement of the glenohumeral joint. Therefore, achieving bone union should be prioritized in the mid-term. On the other hand, further study is warranted to clarify the long-term progression of bone resorption, which may lead to exposure of screws, thereby increasing the risk of screw breakage or irritation.

Complications following coracoid transfer surgery are mostly related to the bone block and the metallic screw, suggesting that it is preferable to employ methods that do not require a bone block or metallic screw. Indeed, soft-tissue Bankart repair has been the standard surgical strategy for managing shoulder instability, though unsatisfactory results continue to be reported despite substantial advancements including tendon autograft reinforcement [21] and conjoint tendon transfer [22]. Bankart repair is reportedly ineffective in patients with severe glenoid bone loss [3], in collision athletes [1, 2, 5–7], in patients with capsular laxity [8], and in patients requiring revision surgery [23]. Coracoid transfer is necessary in such patients. However, further measures should be taken to minimize the risk of complications of coracoid transfer surgery, including bone resorption in the coracoid graft. The present study was motivated by the need to understand the extent and impact of postoperative changes reflecting bone metabolism in the coracoid graft.

This study has several limitations. First, because we used CT imaging, it remains unclear whether the changes visible in the CT images accurately reflect actual bone shaping and resorption as they occurred in the body. Second, the methodology used the position and angle of the screw as the reference for superimposing and classifying the slices, which did not account for possible loosening or misalignment of the screw. However, since screw misalignment was not evident on any of the two CT scans taken for each shoulder, and there were no other findings indicating significant screw loosening, we believe that the measurements are accurate, although the lack of specific measurements of screw loosening does represent a limitation of the research. Third, the number of patients included in the study is small.

## Conclusions

Postoperative bone formation and bone resorption in the coracoid process grafted during the modified Bankart and Bristow procedure depend on whether bone union occurs and on the location of the bone union. Failure to achieve bone union is associated with significantly poorer clinical outcomes. Further study is needed to establish the optimal surgical strategy for achieving bone union in the superior part of the coracoid graft, as well as to clarify the impact of postoperative graft changes on the long-term clinical results.

## Abbreviations

BB: Bankart and Bristow
CT: Computed tomography
